# Bio-psychosocial determinants of time lost from work following non life threatening acute orthopaedic trauma

**DOI:** 10.1186/1471-2474-11-6

**Published:** 2010-01-05

**Authors:** Fiona J Clay, Stuart V Newstead, Wendy L Watson, Joan Ozanne-Smith, Roderick J McClure

**Affiliations:** 1 Monash University, Accident Research Centre, Clayton, Victoria, 3800, Australia; 2 NSW Injury Risk Management Research Centre, University of NSW, Sydney, NSW, 2052, Australia; 3 Monash University, Department of Forensic Medicine, Victorian Institute of Forensic Medicine, Southbank, Victoria, 3006, Australia

## Abstract

**Background:**

To determine factors predicting the duration of time away from work following acute orthopaedic non life threatening trauma

**Methods:**

Prospective cohort study conducted at four hospitals in Victoria, Australia. The cohort comprised 168 patients aged 18-64 years who were working prior to the injury and sustained a range of acute unintentional orthopaedic injuries resulting in hospitalization. Baseline data was obtained by survey and medical record review. Multivariate Cox proportional hazards regression analysis was used to examine the association between potential predictors and the duration of time away from work during the six month study. The study achieved 89% follow-up.

**Results:**

Of the 168 participants recruited to the study, 68% returned to work during the six month study. Multivariate Cox proportional hazards regression analysis identified that blue collar work, negative pain attitudes with respect to work, high initial pain intensity, injury severity, older age, initial need for surgery, the presence of co-morbid health conditions at study entry and an orthopaedic injury to more than one region were associated with extended duration away from work following the injury. Participants in receipt of compensation who reported high social functioning at two weeks were 2.58 times more likely to have returned to work than similar participants reporting low social functioning. When only those who had returned to work were considered, the participant reported reason for return to work " to fill the day" was a significant predictor of earlier RTW [RR 2.41 (95% C.I 1.35-4.30)] whereas "financial security" and "because they felt able to" did not achieve significance.

**Conclusions:**

Many injury-related and psycho social factors affect the duration of time away from work following orthopaedic injury. Some of these are potentially modifiable and may be amenable to intervention. Further consideration of the reasons provided by participants for returning to work may provide important opportunities for social marketing approaches designed to alleviate the financial and social burden associated with work disability.

## Background

One of the most important predictive characteristics of return to work (RTW) following injury is the duration of sick leave taken post-injury [1,2]. Lessons learnt from the natural history of lower back pain are that the longer a person remains off work following injury, the higher the risk of ongoing work disability [2]. Early and durable RTW is a desired outcome of occupational rehabilitation and injury compensation systems [3].

While substantial research has been directed towards musculoskeletal injuries resulting from cumulative trauma, there has been little research examining factors predicting the duration of time away from work following acute trauma. Acute orthopaedic trauma of all severities is a common reason for hospitalization and is often associated with ongoing pain and disability [4-6]. In the small number of studies that address this injury population; higher education, white collar work, high self efficacy and strong social support were associated with earlier RTW while the receipt of compensation and the presence of depression or post traumatic stress were associated with extended time off work [7-9]. However, a number of these studies have had small sample sizes of no more than 60-80 participants [10-12] or considered factors related to time to return to functioning rather than time to RTW [13]. Surprisingly, there has been little consideration in analyses of the reason's stated by workers for returning to work.

The aim of this study was to identify factors that influence the time to RTW in a sample of participants, both compensated and not compensated, who had been hospitalized following acute non life threatening orthopaedic trauma resulting in a range of injuries. The focus was on factors measured early in the course of recovery and that may be potentially amenable to intervention. The study hypothesis is derived from a bio-psychosocial model approach to musculoskeletal work disability; that postulates that factors related to the person, their psychosocial functioning and their environment are as important as physical factors relating to the injury in determining duration of time off work [14]. Finally, for participants who returned to work during the course of the six month study, the study examined whether reasons stated by participants for returning to work were associated with an increased probability of early RTW.

## Methods

### Study design and setting

The Determinants of Outcome in Orthopaedic trauma (DOOT) study is a multi-centre prospective follow-up cohort study conducted in the state of Victoria, Australia.

### Patients and Procedure

Patients presenting to one of four hospitals in Victoria as a result of sustaining acute orthopaedic trauma were recruited to the project. Study hospitals were selected to achieve a representative sample of all people of working age admitted to Victorian public hospitals annually as a consequence of sustaining acute unintentional trauma following an analysis of the Victorian Admitted Episodes Dataset [15]. The VAED is a dataset of acute patient hospital admissions representing 100% coverage of hospital admissions to public hospitals in Victoria.

Hospitals were selected in different geographical regions that broadly reflected a range of socioeconomic status in patients admitted to hospitals. The choice of hospitals was also based on their trauma status under the Victorian State trauma system in order to facilitate the recruitment of patients with a range of orthopaedic injuries. They included a regional hospital, two metropolitan hospitals and a level 1 major trauma hospital. The Level 1 trauma hospital receives more serious injuries or concerning presentations.

The sample inclusion criteria were people aged 18 to 64 who were employed for a wage during the four weeks prior to the injury with English language skills sufficient to allow completion of questionnaires. Patients were excluded if they had sustained an intentional injury, were not employed, or if medical staff considered them to be medically unfit to provide informed consent. Patients with a significant traumatic brain injury associated with prolonged loss of consciousness were excluded because of the documented cognitive sequelae that are not comparable to other types of injury.

Injury factors were retrieved from the patient medical record in order to allow for the coding of the injury according to the Abbreviated Injury Scale (AIS) [16] and the subsequent calculation of the Injury Severity Score (ISS). Patients were classed as having a minor injury if they had an ISS 1-8, moderate injury ISS: 9-15 and a major injury ISS > 15 [17]. The AIS coding was also used to create categories of orthopaedic injuries according to the site of injury.

Patients were recruited following presentation to the hospital emergency department as a result of their injury. Following informed consent, collection of demographic and occupation data together with a retrospective assessment of pre-injury health was conducted at recruitment and patients were further surveyed by phone or in person if they were still in hospital, 2 weeks, 12 weeks and 6 months following their injury. All patients were recruited and followed up between March 2005 and October 2006

### Study Factors

Factors used in the analysis were chosen with respect to the hypothesis being tested and reflected findings from the literature as well as discussions with key informants.

The possible predictors of outcome were grouped as follows.

1. Demographic factors: Age and gender.

2. Pre-injury health: A history of prior pain and the presence of co-morbid health conditions at entry to the study.

3. Injury factors: The main factor was the Injury Severity Score. Other factors were the presence of an orthopaedic injury to more than one region and whether the injury required initial surgery.

4. Occupation factors. Work category (full time or part time), blue collar worker and self employment.

5. Psychosocial factors: Education, compensation status; reported pain levels post injury and psychosocial factors including pain attitudes, recovery expectations and the presence of psychological distress in the form of depression, anxiety or stress.

Pain intensity was measured at the two week follow-up using the short form McGill Pain Questionnaire, a validated dedicated pain measurement tool [18]. Participants were asked to rate their overall pain since the injury according to a six item adjectival scale. The scale is scored: no pain, mild, discomforting, distressing, horrible, and excruciating [18]. Responses were dichotomised into the following groups (high: distressing/horrible/excruciating versus mild: no pain/mild/discomforting).

Compensable status was measured by asking participants if they were receiving injury compensation from state based compensation authorities responsible for work injury and transport related injury. Self employed workers and Commonwealth Government employees are not covered for work related injury under the state based workers compensation scheme [19].

Recovery beliefs were measured by asking the participants at the two week follow-up to rate on a scale from 0-10 the extent to which they believed they would recover enough to return to their usual pre-injury activities [20]. High scores represented strong recovery beliefs. The scores were skewed in the direction of high scores. In order to give sufficiently large cell counts for statistical power, the variable was dichotomised such that scores from 8-10 reflected strong recovery beliefs and scores from 0-7 low to medium beliefs.

A single item question on pain, as it relates to work was adapted from the Survey of Pain attitudes (SOPA), a validated instrument that assesses the impact of patients' feelings about pain control, solicitude, medication, disability, emotion, medical cure and harm [21,22]. Participants were asked at the two week follow-up if they agreed or disagreed with the statement that they shouldn't work with their current level of pain. Possible responses were 1. Strongly agree 2. Moderately agree 3. Slightly agree 4. Slightly disagree, 5. Moderately disagree 6. Strongly disagree. Responses were dichotomised agree (1-3) vs. disagree (4-6).

Co-morbid health conditions were obtained from the medical records pertaining to the current injury. Age was assessed for the effect of each year as a continuous variable.

The social functioning scale from the 36 item Short Form (SF36) Health Survey; a validated generic questionnaire that examines health related functioning and well being from the patient's viewpoint was used to examine social functioning at the two week follow-up [23,24]. The SF36 has been demonstrated to be comprehensive and psychometrically robust in many studies and has been validated in an injury population [24,25]. The scale includes two questions which measure the impact of the injury on normal social activities [23]. The scale is scored from 0-100 with higher scores reflecting better scores. Raw scores were dichotomised such that scores above 75 reflected high social functioning and scores from 0-74 low to moderate social functioning.

Negative emotional states of depression, anxiety and stress were assessed using the DASS21 a generic measure comprising three self-report scales [26]. These measures were collected retrospectively to establish pre-injury baselines and prospectively at the two weeks follow-up. The scales were scored and categorised according to the recommended cut-offs [27]. A composite variable (normal versus psychological distress) was created in which participants who reported symptoms of depression, anxiety or stress regardless of severity was grouped together and participant's whose responses were categorised as normal for depression, anxiety and stress were similarly grouped.

Participants who had returned to work during the study were asked to state the reasons for their RTW. A list of options was provided and participants were able to provide other responses. More than one response was permitted. The most common responses were dichotomised (e.g "to fill the day" vs. all alternate responses) and considered as factors in the multivariate analysis of factors associated with the time of RTW in those who returned to work during the study period.

Variables with multiple categories were dichotomized for analysis to give sufficiently large cell counts for adequate statistical power in the multivariate analysis. Dichotomous categories defined for each factor were: educational status (completed university degree versus completed less than university degree, ISS scores (ISS<9 versus ISS>= 9) based on the common groupings for minor, moderate and major trauma and co-morbid health conditions (none versus one or more).

### Assessment of Return to Work outcomes

The main outcome in the analysis was the time (in days) until first RTW on either full duties or modified work following the injury event. At each follow-up time point, participants were asked if they had returned to work. Participants who answered in the affirmative were asked on what date they returned to work. If a participant who had returned to work reported that they were doing the same duties and hours as they had been prior to the injury, this was recorded as full duties. If a participant reported that they were working modified hours and/or performing different or a reduced number of tasks as a result of their injury, this was recorded as modified work.

The time variable was constructed by subtracting the RTW date provided by the participant from the date of the injury as indicated by the medical records. Ninety-two of the 104 participants who returned to work provided the exact date of return; the remaining participants were only able to indicate the month that they returned. In those cases, the midpoint of the month was used as the date of first RTW in order to minimise possible error. The participants own statement of return to work was not validated from other sources.

The second outcome under consideration included only those who had returned to work by six months and, as such, there was no censoring due to non return to work or loss to follow-up.

### Statistical analysis

Kaplan-Meier estimate analyses were used to plot the cumulative proportions of study participants returning to work as a function of the duration of time off work and to calculate the median time to RTW for the whole group. This was done for each of the independent risk factors, one factor at a time. The Log Rank test and Breslow tests were used to test the equality of survival functions [28,29]. Visual inspection of graphical output was used to assess whether levels of each factor violated the proportional hazards assumption.

Cox Proportional Hazard (PH) regression analysis was used to examine the combined effect of personal, occupational, injury and psychosocial factors on the duration of time off work. Subjects were right censored if lost to follow-up or if they did not RTW during the six month study period.

In order to reduce the potentially large number of factors included in the multivariate models, in the first instance the relationship between the study factors and the outcome of interest was examined using univariate Cox (PH) regression analyses. Variables were included in the multivariate model if both the *P *value from the univariate Cox regression analysis and the *P *value from the test of equality of survival functions were statistically significant at p <= 0.15. Age as a continuous variable was included in the multivariate model irrespective of the level of significance. Correlation coefficients were also calculated for all independent variables considered for the model. If a correlation >0.35 was found between a pair of determinants, one factor was removed from the analysis. All remaining factors of interest were entered simultaneously into the model. Potential confounders were then included in the model one at a time. Factors that violated the proportional hazards assumption were treated as time dependent covariates if they satisfied the criteria for use in the model or included as stratifying variables if they did not.

In the analysis of those participants who returned to work during the study, all study factors were entered simultaneously into the model. The reasons stated by participants for RTW were then tested in the model one at a time regardless of the level of significance achieved in the univariate analysis. Only RTW reasons that achieved a statistical significance at p = 0.05 or changed the relative rate ratios by more than 10% remained in the final model. Potential confounders were then tested in the model one at a time.

A confounding factor was retained in the model if it was a statistically significant predictor of the outcome and the hazard rates of other factors in the model were changed by >10%. If the potential confounder was not significant but the hazard rates were changed by >10% the two models were compared and a decision was made on whether to retain the variable in the final model. The decision was based on whether or not the confounder changed the overall conclusions for the final model. Two way interactions between study factors were tested by adding interactions to the model one at a time. Interactions were retained in the final model if they improved the log likelihood of the model with a statistical significance of *p *< 0.05 based on the chi-squared test of model fit improvement. Potentially influential outliers were assessed by visual inspection of graphical output of plotted *DfBeta *variables. If the removal of the outlier resulted in considerable change to the effect sizes of other factors in the model represented by the model parameters or changed the parameter significance levels and hence the overall conclusions, then the outlier was removed from the final model reported.

The Hazard ratios reported in the regression analyses are presented as a relative RTW rate ratio (RR) with 95% confidence intervals (95% C.I). The RR provides an estimate of the relative likelihood of RTW at any given time after the injury. A RR below 1 means the factor is associated with extended time off work relative to the reference category. A RR > 1 means the factor is associated with reduced time off work. A significance probability (*P*) values of < 0.05 was considered significant. The SPSS software (Version 15.0, SPSS Inc, USA) was used for all statistical analyses.

### Ethics

Ethics approval was obtained from the Standing Committee on Ethics in Research Involving Humans of Monash University and the corresponding ethics committees at all participating hospitals.

## Results

### Demographics and Injury characteristics

**Figure 1:**
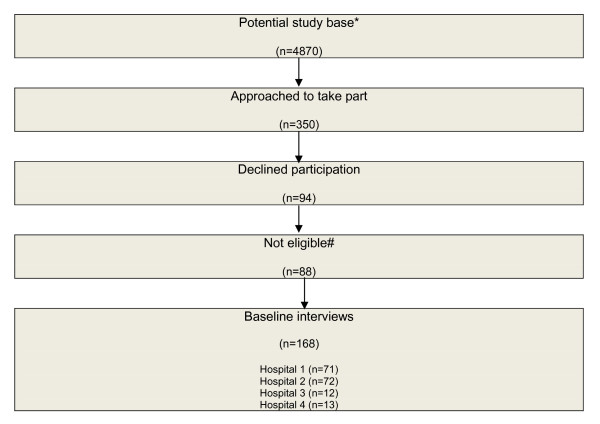
**Flow chart documenting study recruitment**. (*Includes persons not employed and persons with English language skills that would preclude participation in the study. #According to inclusion and exclusion criteria)

One hundred and sixty eight patients were recruited to the study and completed baseline surveys. The numbers of potentially eligible subjects, refusals, and participants are shown in Figure 1. Information on RTW status at six months was available for 152 participants (90.4%). The mean age of the sample was 37.7 years and the cohort consisted primarily of men (75%). Using the ISS to classify injuries; 88 patients sustained minor injuries (ISS 1-8), 69 moderate (ISS 9-15) and 11 major injuries (ISS>15). The majority of orthopaedic injuries sustained were isolated or multiple injuries to the lower or upper extremities (73%). Eleven percent of the sample sustained both orthopaedic and non orthopaedic injuries. For 77% of the participants, their injury included a fracture. Other orthopaedic injuries sustained by participants included dislocations, lacerations, tendon tears, and partial or complete amputation of fingers or toes.

At the end of the follow-up period 104 participants had returned to work and 48 participants remained off work. Sixteen participants were lost to follow-up and were coded as censored cases for the analyses. Unadjusted Kaplan-Meier estimates of the cumulative proportion of study participants returning to work at 3, 4.5 and 6 months post injury were 0.44, 0.60 and 0.68 respectively. The median time to RTW was 97 days (SD: 11.71). Of those who returned to work, 56% returned first to modified work. There was no significant difference in the duration of time off work between those who returned to modified work and those who returned to full duties.

Cumulative proportions returning to work by 6 months post injury were 0.78 for ISS minor injuries versus 0.53 for participants sustaining injuries with an ISS>9 (Table 1). The median time to RTW was 77 days for ISS 1-8 and 150 days for ISS>8. For non compensated patients, the median time to RTW was 64 days and 146 days for participants in receipt of injury compensation. The difference in overall time to recovery for ISS 1-8 versus ISS>9 and for compensated versus non compensated participants was statistically significant (p = 0.01).

**Table 1:** Univariate and Kaplan-Meier Estimates of the Cumulative Proportions of RTW by participant characteristics

			Proportion with First RTW by			Univariate analysis		
			
		N	3 months	4.5 months	6 months	*P*	Relative Rate Ratio	95% C.I
Gender	Male	126	0.42	0.56	0.66	0.130	1.38	(0.90-2.10)
	Female	42	0.56	0.70	0.78			
Age	18-34 yrs	70	0.42	0.56	0.67			
	35-44 yrs	44	0.53	0.61	0.66	0.645	1.12	(0.69-1.81)
	45-64 yrs	54	0.44	0.63	0.65	0.948	0.98	(0.62-1.55)
Education*	Less than University	136	0.43	0.56	0.63	0.374	1.23	(0.77-1.96)
	University	32	0.58	0.74	0.77			
Shouldn't work with current pain level*	Disagree	52	0.61	0.75	0.79	0.001	0.48	(0.32-0.72)
	Agree	103	0.34	0.50	0.59			
Self employed worker *	No	145	0.43	0.56	0.63	0.005	2.14	(1.24-3.64)
	Yes	23	0.83	0.90	0.90			
Initial pain intensity*	Low	104	0.52	0.68	0.72	0.021	0.61	(0.41-0.93)
	High	64	0.36	0.47	0.58			
Psychological distress	No	94	0.50	0.60	0.66	0.719	0.93	(0.62-1.37)
	Yes	67	0.40	0.57	0.68			
Receipt of Compensation*	No	72	0.58	0.74	0.79	0.002	0.53	(0.36-0.79)
	Yes	91	0.37	0.41	0.57			
Co-morbid health conditions*	No	112	0.51	0.67	0.71	0.036	0.62	(0.40-0.96)
	Yes	56	0.36	0.47	0.57			
Injury severity score*	1-8	88	0.54	0.70	0.78	0.001	0.51	(0.34-0.76)
	>9	80	0.36	0.48	0.53			
Orthopaedic injury: > than 1 region*	No	137	0.52	0.65	0.71	0.007	0.45	(0.25-0.80)
	Yes	31	0.20	0.38	0.45			
Recovery beliefs*	High	124	0.51	0.63	0.69	0.118	1.47	(0.90-2.41)
	Low	37	0.33	0.48	0.56			
Blue Collar Worker*	No	81	0.54	0.67	0.76	0.020	0.62	(0.42-0.93)
	Yes	87	0.37	0.51	0.56			
Initial surgery required*	No	49	0.54	0.72	0.79	0.012	0.59	(0.39-0.89)
	Yes	118	0.42	0.54	0.60			
Pain prior to injury	No	128	0.45	0.58	0.64	0.479	1.17	(0.75-1.85)
	Yes	34	0.57	0.64	0.73			
Social functioning*	High	44	0.66	0.74	0.79	0.001	2.02	(1.33-3.07)
	Low	115	0.38	0.54	0.61			

*Significant p < 0.15 (Log Rank Test/Breslow test) The data in the table do not refer to either the Log Rank or Breslow test.

Univariate analysis showed characteristics of the participants associated with earlier RTW included higher social functioning at 2 weeks post injury and self employment. Factors associated with a slower RTW included injury factors, psychosocial factors and job characteristics. Receipt of compensation, negative pain attitudes with respect to work, injury severity, initial need for surgery, orthopaedic injury to more than one region, co-morbid health conditions at study entry and blue collar work were all associated with increased time off work. Age, gender, education, prior pain and psychological distress at two weeks post injury were not associated with time to RTW in univariate analysis.

In the final multivariate Cox (PH) model, after adjustment for gender; the presence of co-morbid health conditions, high initial pain intensity, older age, negative pain attitudes in relation to work, blue collar work, an orthopaedic injury to more than one body region and an injury that required initial surgery were all associated with increased time off work. High recovery beliefs, injury severity, and age did not reach statistical significance. Results of the Cox regression analysis are reported in Table 2.

**Table 2:** Significant Independent Predictors (Adjusted) of the Relative Rate Ratio of RTW during the six month study

Main effects model only			
**Factor**	***P***	**Relative Rate Ratio** **(adjusted)**	**95% C.I**

Should not work with current pain: agree	0.007	0.54	0.35-0.84
Co-morbid conditions: one or more	0.027	0.59	0.37-0.94
Blue collar worker	0.016	0.56	0.35-0.89
Orthopaedic injury: more than one region	0.030	0.49	0.26-0.93
Social functioning: high	0.009	1.89	1.17-3.07
Injury severity: ISS >9	0.036	0.36	0.39-0.97
Age (cont)	0.017	0.17	0.96-0.99
Self employed	0.015	2.31	1.18-4.53

**Main effects plus interactions**			

**Factor**	***P***	**Relative Rate Ratio** **(adjusted)**	**95% C.I**

Should not work with current pain: agree	0.002	0.49	0.31-0.77
High Initial pain intensity	0.008	0.47	0.27-0.82
Co-morbid conditions: one or more	0.016	0.56	0.35-0.89
Blue collar worker	0.008	0.52	0.32-0.84
Initial need for surgery	0.034	0.61	0.39-0.96
Orthopaedic injury: more than one region	0.007	0.41	0.21-0.78
Injury severity: ISS>9	0.045	0.63	0.39-0.99
Age (cont)	0.037	0.98	0.96-0.99
			
Education*high initial pain level	0.006	4.50	1.55-13.09
Relative rate ratio for interaction	0.56 × 0.47 × 4.50 = 1.18		
Social functioning* receipt of compensation	0.010	3.58	1.35-9.47
Relative rate ratio for interaction	1.22 × 0.59 × 3.58 = 2.58		

Factors included in the final main effects model. Shouldn't work, age, education, injury severity, compensation, blue collar worker, co-morbidities, orthopaedic injury to more than one region, high initial pain, recovery beliefs, self employment. Confounding Factors tested but did not remain in the final model: gender, prior pain

Interactions were tested and two interactions met the criteria to remain in the model. In the first interaction (social functioning by receipt of compensation), the reference category was those who had low social functioning and were not receiving compensation. The interaction indicated that the effect of compensation status was not consistent across different levels of social functioning. The addition of the interaction complicates interpretation of the coefficients of the main effects of the terms comprising the interaction. Participants who reported high social functioning at two weeks post injury and were in receipt of injury compensation had a 2.58 times increased rate of RTW when compared to participants in receipt of compensation who reported low social functioning. In the second interaction (education by initial pain levels), the reference category was those who were educated to less than University level and reporting low initial pain levels at two weeks post injury. Participants reporting high initial pain levels who were educated to university level had marginally less time away from work (Relative Rate ratio: 1.13) than participants reporting high initial pain but educated to less than University.

### Reasons for Return to Work

Of those who went back to work, the median time to RTW for participants who reported that it was "because they felt able to" was 72 days versus 58 days for all other reasons. Those who reported "financial security" as the reason for RTW returned in a median time of 54 days versus 64 days for other responses. The difference in the median time to RTW was not statistically significant for either reason. Participants who indicated they went back to work in order "to fill their day" showed a statistically significant difference in time to recovery (log rank: p = 0.036, data not shown) when compared to participants who reported other reasons for returning to work. The median time to RTW was 45 days for those who needed "to fill their day" versus 60 days for other responses. Ninety three percent of participants who reported the need "to fill the day" were back at work by three months compared to 66% who indicated other reasons.

In univariate analysis, characteristics associated with less time off work included, self employment, the need "to fill the day" and higher social functioning whereas characteristics associated with slower RTW included high initial pain intensity, co-morbid health conditions and psychological distress. Injury severity, compensation, age, gender and education were not associated with time off work for participants who returned to work within 6 months (data not shown).

In the final Cox PH analysis when considered in the context of other factors in the model, factors associated with slower RTW in those that returned to work within 6 months included high initial pain intensity, co-morbid health conditions and the need "to fill the day". Financial security and "because I feel able to" were individually tested in the model but did not fulfil the criteria to remain in the model. When "to fill the day" was added to the model, it was a significant predictor of less time off work [ARR 2.19 (95% C.I: 1.23-3.88)]. To fill the day showed evidence of a confounding effect on both co-morbid health conditions and high initial pain intensity. Results of the Cox regression analysis are reported in Table 3.

**Table 3:** Significant Independent Predictors (Adjusted) of the Relative Rate Ratio of RTW for participants who returned to work during the 6 month study.

Main effects model only			
**Factor**	***P***	**Relative Rate Ratio** **(adjusted)**	**95% C.I**

Co-morbid conditions: one or more	0.001	0.52	0.33-0.84
To Fill the day	0.003	2.19	1.23-3.88
High Initial pain intensity	0.022	0.57	0.35-0.92

**Main effects plus interaction**			

**Factor**	***P***	**Relative Rate Ratio** **(adjusted)**	**95% C.I**

Co-morbid conditions: one or more	0.001	0.44	0.26-0.72
To Fill the day	0.003	2.41	1.35-4.30
High Initial pain intensity	0.008	0.51	0.31-0.84
Co-morbid conditions* self employment	0.009	7.14	1.62-31.48
Relative rate ratio for interaction	0.44 × 0.84 × 7.14 = 2.63		

Factors included in the final main effects model (Shouldn't work, social functioning, psychological distress, age, orthopaedic injury to more than one region, self employment, co morbidities, initial pain intensity, self employment. Study factors tested that did not remain in the final model: financial security, because I feel able to. Confounding factors tested but did not remain in the final model: gender, education, injury severity

Interactions were tested and one interaction (co-morbid health conditions by self employed worker) met the criteria to remain in the model. For this interaction, the reference category was those who reported no co-morbid health condition and were not self employed. The interaction indicated that the effect of co-morbidities was not consistent across different types of work. Participants who reported co-morbidities at study entry and were self employed had 2.63 times less time off work than similar participants who worked for an employer.

Potential influential observations were again assessed by plotting *DfBeta *variables. One observation was identified as potentially influential and removed from the analysis and the model rerun after first ensuring that the case did not reflect a data entry or coding error. As the removal of the observation did not significantly change the effect sizes of other factors in the model or the conclusions drawn from the model, the final model includes all observations.

## Discussion

To date, the focus of most research on disability following acute orthopaedic trauma has been on outcomes following major trauma, severe life threatening injuries or specific injuries [30,31]. There has been a relative lack of research addressing minor or moderate injuries despite the knowledge that these injuries are common and contribute significantly to the burden of injury both with respect to short term as well as lifetime morbidity [32,33]. The current study was designed to establish determinants of the duration of time off work following a range of non life threatening orthopaedic injuries requiring hospitalization. The findings confirm that injuries of both minor and moderate severity are associated with extended time away from work. In this study, the cumulative proportions of RTW by six months are 0.78 for minor injuries and 0.53 for injuries of moderate or higher severity. During the six-month study period, 68% of participants were able to RTW; of those, 56% returned to modified work as a result of ongoing injury related limitations.

The significant social and financial costs to the injured person and their family as well as the costs to the employer of replacing a worker off work as a result of an injury (regardless of where it occurred or its level of severity) underscore the public health impact of these injuries in terms of lost work days as well as the costs associated with ongoing rehabilitation. Together, they highlight the need for ongoing research into factors that effect the duration of time off work.

Many of the determinants identified in this study including blue collar work and older age are consistent with other studies both in acute and cumulative trauma populations [7,34,35]. These are further outlined below. In common with other studies, gender was not a significant predictor of the duration of time off work. Our study sample included a similar proportion of males to these studies [8,9]. Other factors that were not measured including job demands, post traumatic stress, and income may also impact on the extent of time off work. The rate of RTW observed in this study is consistent with the findings from other studies that include similar injuries and the same follow-up periods [8].

A number of studies have found that pre-injury medical conditions are associated with ongoing disability following acute trauma [36]. In the current study, pre-injury co-morbid health conditions were associated with delayed RTW. One third of our sample reported at least one health condition at study entry including obesity, cancer, illicit drug use, and depression or anxiety. These conditions may affect the recovery process by limiting the person's ability to physically or psychologically engage with rehabilitation programs. The finding of an interaction between pre-injury co-morbidities and self employment is worth noting. Participants reporting co-morbid health conditions who were self employed had a 2.63 times increased rate of RTW when compared to similar participants who worked for an employer. Self employed workers have limited entitlements under the Victorian workers compensation scheme and unless they have some form of income protection insurance coverage would only be eligible for limited wage replacement through the social security system in the event of extended disability [19]. The lack of insurance coverage may result in self employed workers returning to work earlier regardless of the nature of their injury or the presence of pre-injury co-morbidities. The effect of this on longer term health and disability has not been studied.

In the current study, we found that a number of injury related factors including injury severity, the initial need for surgery and an orthopaedic injury to more than one region were associated with extended time off work. These findings are consistent with other studies that have shown that higher scores indicating more severe injuries on either the Hand Injury Severity Scale or the Modified Hand Injury Severity Scale are associated with extended time off work [12,37]. An injury that required initial surgery was also associated with extended time off work in the current study. This factor has received little attention in the literature although in a study of factors that predicted days of total disability following a work-related traumatic amputation, the number of surgical procedures was a significant predictor of more days of total disability [38].

There were a number of significant interactions between study factors that provide additional insight into the effects of factors of importance. Systematic reviews have noted the strong effect of high initial pain intensity on disability [39]. Although the mechanism by which high initial pain leads to disability is unknown, it is possible that it initiates a set of behaviours that result in the affected person being more prone to psychological distress. This study provides support for the role of a higher level of education marginally attenuating this effect. The study also found that negative pain attitudes with respect to work as measured by a single item adapted from the survey of pain attitudes (SOPA) was associated with extended time off work. The SOPA developers report that people who demonstrate negative pain attitudes with regards to belief in ones ability to function because of pain are more likely to experience ongoing disability [22].

The receipt of compensation has been noted as a strong correlate of extended work disability in many studies [40] although in this and other studies it was not a significant predictor of outcome. In univariate analysis, the receipt of compensation was associated with slower recovery. However, when considered in the context of other factors, the receipt of compensation did not achieve statistical significance (p = 0.052). The results suggest that this factor may be important and worthy of further study but that the analysis lacked sufficient power to detect a significant effect. While only 54% of study participants received some injury compensation, we did not establish what that entailed and only a small number of participants indicated that they were pursuing litigation.

In the current study, high social functioning (SF-36) measured at two weeks post injury was associated with an increased rate of RTW. Social functioning may reflect social support both with respect to the home and work as well as personal coping skills including self efficacy. Similar findings have been observed in a study of RTW after severe multiple injuries although in this study social functioning was measured at two years post-injury [31]. The findings from our study suggest that the measurement of social functioning at an early stage in recovery may provide valuable insight into longer term recovery. Interpreting the significant interaction between social functioning and compensation status showed that the effect of high social functioning was attenuated by the receipt of compensation such that the relative rate of RTW was increased in those who reported high social functioning and were in receipt of compensation. It suggests that the receipt of compensation may have more of a negative effect on RTW in those that do not report high social functioning. This finding warrants follow-up in future studies.

Survival analyses of factors predicting time to RTW commonly considers both participants who have returned to work together with participants who are censored because they did not RTW or were lost to follow-up. In a secondary analysis, we restricted our sample to only those participants who went back to work in order to determine whether the reasons provided by participants for returning to work were significant predictors of outcome when considered in the context of other factors. As far as we are aware, no study has considered reasons provided by injured workers for RTW in multivariate analyses. Those participants who went back to work in order "to fill their day" returned 2.41 times faster than participants who provided alternate responses after adjusting for potential confounding. Neither financial security nor "because they were able to" predicted the duration of time off work. Our findings concur with clinical impressions by treating providers as well as studies on the role of work that conclude that work provides a sense of purpose to the day by providing structure, activities and an opportunity to be part of something [41]. The findings highlight the need for further consideration of reasons provided by injured workers as they are potentially amenable to targeting using social marketing approaches. Social marketing has been used as an effective strategy for delivering preventive health messages including changing societal views on back pain [42].

The results of the study should be interpreted in light of a number of strengths and limitations. The strengths of this study include its prospective longitudinal design, high follow-up rate and that only ten percent of cases were censored due to missing data. The study is limited by its small sample size which limits the number of factors that can be assessed and the levels within these factors that can be considered discretely and also affects the power of the study and its ability to detect effects. The heterogeneous nature of injuries means that conclusions with respect to particular injury types can only be limited. Twelve percent of those who went back to work were only able to provide the month that they returned and we were not in a position to validate this from other sources. As such this has the potential to introduce bias; however the use of the midpoint of the month for these participants should limit the extent of any bias. Other potential limitations involve the use of an individual item from the SOPA as a marker of pain attitudes as this approach has not been validated in an orthopaedic trauma population. Finally, while the retrospective measurement of pre-injury health is considered important as it allows for comparison by which to assess individual recovery; the potential for recall bias to affect the person's view of their pre-injury health must be acknowledged. As long as the assessment is made as soon after the injury event occurred as practicably possible, this approach is considered reasonable [43].

## Conclusions

This study has demonstrated the importance of a number of injury related and psychosocial factors on the rate of RTW following acute non life threatening orthopaedic trauma. The number of factors predictive of time away from work highlights that RTW following injury represents a complex and multifactorial process and presents potential challenges with respect to how to address the problem. A number of the significant determinants of outcome are potentially amenable to intervention. Of those participants who had returned to work, those that reported the need "to fill the day" returned to work earlier. An increased understanding of the determinants of time to RTW and the reasons for RTW from the viewpoint of the injured worker is essential to understanding the complexity of the RTW process, improving functional outcomes and reducing the social and financial burden associated with injuries.

## Competing interests

The authors declare that they have no competing interests.

## Authors' contributions

FC conceived of the study, participated in its design and coordination, was responsible for the acquisition of data, participated in the design of the statistical analysis, performed the statistical analysis, analysed and interpreted the data and prepared the manuscript. SV participated in the design of the statistical analysis and the analysis and interpretation of the data and in review of the manuscript for important intellectual content. WW participated in the design and coordination of the study. JOS participated in the design and coordination of the study. RM participated in the coordination of the study, the analysis and interpretation of the data and in the review of the manuscript for important intellectual content. All authors read and approved the final manuscript.

## Pre-publication history

The pre-publication history for this paper can be accessed here:

http://www.biomedcentral.com/1471-2474/11/6/prepub
